# Expression of intelectin-1 in bronchial epithelial cells of asthma is correlated with T-helper 2 (Type-2) related parameters and its function

**DOI:** 10.1186/s13223-017-0207-8

**Published:** 2017-08-01

**Authors:** Taiji Watanabe, Kazuyuki Chibana, Taichi Shiobara, Rinna Tei, Ryosuke Koike, Yusuke Nakamura, Ryo Arai, Yukiko Horigane, Yasuo Shimizu, Akihiro Takemasa, Takeshi Fukuda, Sally E. Wenzel, Yoshiki Ishii

**Affiliations:** 1 Department of Pulmonary Medicine and Clinical Immunology, Dokkyo Medical University School of Medicine, Tochigi, Japan; 2 Dokkyo Medical University School of Medicine, 880 Kitakobayashi Mibumachi, Shimotsugagun, Tochigi, 321-0293 Japan; 3 Pulmonary Allergy and Critical Care Medicine, Department of Medicine, University of Pittsburgh, 3459 Fifth Ave., Pittsburgh, PA 15213 USA

**Keywords:** Intelectin-1, Bronchial asthma, Bronchial epithelial cells, IL-13, Type-2 related parameters

## Abstract

**Background:**

Intelectin-1 (ITLN-1) is secreted by intestinal goblet cells and detectable in blood. Its expression is increased in IL-13-overexpressing mouse airways. However, its expression and function in human airways is poorly understood.

**Methods:**

Distal and proximal bronchial epithelial cells (BECs) were isolated from bronchoscopic brushings of disease control (D-CON), COPD, inhaled corticosteroid-treated asthma (ST-Asthma) and inhaled corticosteroid-naïve asthma (SN-Asthma) patients. *ITLN*-*1* mRNA expression in freshly isolated BECs, primary cultured BECs with or without IL-13 and inhibition effects of mometasone furoate (MF) were investigated by quantitative real-time PCR (qPCR). Correlations between *ITLN*-*1* mRNA and Type-2 related parameters (e.g. FeNO, IgE, *iNOS, CCL26, periostin* and *DPP4* mRNA) were analyzed. ITLN-1 protein distribution in asthmatic airway tissue was assessed by immunohistochemistry. Bronchial alveolar lavage (BAL) and serum ITLN-1 protein were measured by ELISA. The effect of recombinant human (rh) ITLN-1 on stimulated production of CXCL10 and phospho(p)-STAT1 expression examined in lung fibroblasts.

**Results:**

*ITLN*-*1* mRNA was expressed in freshly isolated BECs and was correlated with Type-2 related parameters. ITLN-1 protein was increased in goblet cells in SN-Asthmatics and increased in SN-Asthmatic BAL fluid. There were no any differences in serum ITLN-1 concentration between ST and SN-Asthma. IL-13 enhanced ITLN-1 expression and inhibited by MF from BECs in vitro, while rhITLN-1 inhibited CXCL10 production and p-STAT1 expression in HFL-1 cells.

**Conclusion:**

ITLN-1 is induced by IL-13 and expressed mainly in goblet cells in untreated asthma where its levels correlate with known Type-2 related parameters. Further, ITLN-1 inhibits Type-1 chemokine expression.

**Electronic supplementary material:**

The online version of this article (doi:10.1186/s13223-017-0207-8) contains supplementary material, which is available to authorized users.

## Background

Asthma affects nearly 300 million people worldwide but is a heterogeneous disorder comprised of different inflammatory characteristics. Type-2 cytokines (specifically, interleukin (IL)-4, IL-5, and IL-13) are known to play a substantial pathobiological role in many cases. These cytokines, including IL-13 contribute to a Type-2-high molecular asthma phenotype in about 50% of patients with asthma, and are widely believed to play important roles in asthma pathophysiology [1–7]. Furthermore, IL-13-induced periostin [8] and DPP4 can be measured in peripheral blood and are used as biomarkers to predict the efficacy of anti-IL-13 antibodies in human asthma patients [9–11].

Intelectin-1 (ITLN-1) was cloned in 1998 by Komiya et al. from the murine intestinal tract [12]. Human ITLN-1 is a prophylactic soluble lectin discovered that recognizes galactofuranose in the bacterial cell wall [13]. The expression of ITLN-1 in the gastrointestinal tract is strongly induced by parasitic infections [14, 15], suggesting that it is associated with prophylaxis in the gastrointestinal tract. ITLN-1 has been primarily studied in the gastrointestinal tract where it is expressed in intestinal goblet cells, primarily from fetal small intestine. It is detected in blood and can be measured intraluminally as well [16]. ITLN-1 is increased in the airways of IL-13-overexpressing mice, where it appears to be a protein component of mucus associated with intense eosinophilic airway inflammation [17, 18]. However, its expression and role in human asthmatic airways is poorly understood. ITLN-1 was also reported as one of the adipocytokine with anti-inflammatory effects [19]. CXCL10 is a chemokine that attracts T-helper (Th)1 cells [20] and strongly induced by IFNγ. When viral infection occurs, viral recognition receptors, such as Toll-like receptor 3 (TLR3) expressed on BECs, are activated to produce inflammatory cytokines and chemokines, including CXCL10 [21]. Autocrine activation of interferon (IFN) receptors further activates Janus kinase-Signal Transducers and Activator of Transcription (JAK-STAT) signaling pathway, promoting an antiviral state. Moreover, fibroblast like cell produce type I IFN and CXCL10 after stimulation with double stranded RNA [22], perhaps contributing to the pathogenesis of viral infections. Little knowledge exists concerning how the fibroblasts respond to ITLN-1 and which signaling pathways might be involved.

We hypothesized that whether ITLN-1 was induced by IL-13 and correlated to type-2 related markers and inhibited Th1 signaling pathway. In this study, we evaluated *ITLN*-*1* mRNA and protein expression in airway cells, tissue and fluid from asthma, COPD, and disease control subjects obtained via bronchoscopy. BAL and serum ITLN-1 levels were also measured. We compared expression of *ITLN*-*1* mRNA with various Type-2 related parameters. Finally, we investigated a possible function of ITLN-1 in the airways.

## Methods

### Study population

We conducted a retrospective study of 61 patients who visited the Department of Pulmonary Medicine and Clinical Immunology of Dokkyo Medical University Hospital from June 2009 to March 2014 (Table 1). Bronchial brushings were performed to analyze the expression levels of *ITLN*-*1* mRNA. Transbronchial lung biopsy (TBLB) and endobronchial biopsy (EBB) were performed. All subjects met the American Thoracic Society criteria for asthma and had a pre-bronchodialator FEV1 greater than 80% of predicted with an FEV1/FVC greater than 70%. The ST-Asthma group was regularly treated with inhaled corticosteroids (ICS), while the Steroid Naïve (SN)-Asthma group had symptoms, such as cough with wheezing and night time dyspnea, but had not been treated with ICS or oral corticosteroid (OCS) for at least 6 months. Patients were defined as having COPD if the forced expiratory volume in 1 s (FEV1)/forced vital capacity (FVC) (FEV1/FVC) was <70% with fixed bronchial obstruction after bronchodilator. Disease control subjects (D-CON) were defined as those without asthma/COPD who had undergone bronchoscopy because of abnormal chest X-ray shadows. Lung cancer was found in most of D-CON and COPD patients by bronchoscopy. D-CON (as opposed to healthy control) participants were studied, as research bronchoscopy on healthy individuals is not allowed in Japan. Written informed consent was obtained from all participants to perform the procedure and utilize extra tissue/cells for research purposes. This study was approved by the Ethics Committee of Dokkyo Medical University School of Medicine (hop-m22095).

**Table 1:** Total subjects in this study

	N	Age	M:F	FEV1/FVC (%)	FEV1 (%)	FeNO (ppb)	ICS (µg)	OCS use	Smoker (N:E:C)
D-CON	13	61 ± 5***	11:2	78 ± 2	93 ± 3	27 ± 3	0	0	4:8:1
COPD	17	72 ± 2*	14:3	50 ± 4*	57 ± 6	32 ± 7	0	0	1:7:9
ST-Asthma	13	51 ± 4	10:3	73 ± 5	88 ± 6	45 ± 5	723 ± 86	2	3:8:2
SN-Asthma	18	48 ± 4	13:5	73 ± 3	83 ± 3	129 ± 2*	0	0	6:10:2

*p < 0.0001 vs ST or SN-Asthma, ***p < 0.05 vs other groups

### Bronchoscopy with bronchial epithelial cell brushing

Bronchial brushings were performed with a standard, sterile, single-sheathed nylon cytology brush (Olympus T-260; Olympus, Tokyo, Japan). A total of 4 brushings were performed in the distal and proximal airways. Distal bronchial epithelial cells (BECs) were obtained from airways situated about 1 cm away from the pleura, as identified by X-ray guidance [7]. Proximal BECs were collected by scraping directly from the second carina. TBLB and EBB were available from a small number of participants for ITLN-1 expression by immunohistochemistry. We did not collect bronchial alveolar lavage (BAL) and serum samples at the beginning of this study. Given the results of microarray study [7] that strongly expressed *ITLN*-*1* mRNA in SN-Asthma as well as IL-13 stimulated cells, we decided to accumulate serum and BAL samples from asthma patients subsequently. Some cases were not able to collect BAL samples because of severe cough or hypoxemia. Finally, total 18 subjects (5 ST-Asthma and 13 SN-Asthma) were able to collect BAL and 16 subjects (6 ST-Asthma and 10 SN-Asthma) serum samples (Tables 2, 3). Table 4 shows 3 subjects who were received bronchoscopy pre and post ICS-treatment.

**Table 2:** Subjects for analysis of BAL ITLN-1 protein

	N	Age	M:F	FEV1/FVC (%)	%FEV1 (%)	FeNO (ppb)	ICS (µg)
ST-Asthma	5	53 ± 4	4:1	81 ± 3	90 ± 8	53 ± 11	520 ± 120
SN-Asthma	13	51 ± 4	10:3	71 ± 3	82 ± 4	124 ± 23	0

**Table 3:** Subjects for analysis of serum ILTN-1 protein

	N	Age	M:F	FEV1/FVC (%)	%FEV1 (%)	FeNO (ppb)	ICS (µg)
ST-Asthma	6	53 ± 6	3:3	80 ± 5	96 ± 6	42 ± 31	800 ± 120
SN-Asthma	10	43 ± 5	8:2	77 ± 4	86 ± 5	147 ± 24**	0
Healthy control	8	25 ± 1**	5:3	ND	ND	19 ± 2	0

**p < 0.01 vs other groups

**Table 4:** Subjects for analysis of ILTN-1 mRNA pre or post ICS treatment

	N (distal: proximal)	Age	M:F	FEV1/FVC (%)	%FEV1 (%)	FeNO (ppb)	ICS (µg)
ST-Asthma (post treatment)	6 (3:3)	65 ± 4	3:0	69 ± 4	84 ± 4	51 ± 4	400
SN-Asthma (pre treatment)	6 (3:3)	65 ± 4	3:0	68 ± 4	89 ± 4	171 ± 49***	0

***p < 0.05 vs ST-Asthma

### Quantitative real-time PCR

Expression of *ITLN*-*1, iNOS, CCL26*, *periostin* and *DPP4* mRNA in BECs and the expression of *CXCL10* mRNA in HFL-1 cells were following reverse transcription (RT), and then real-time quantitative SYBR Green fluorescent PCR, as described previously [2, 3, 7]. First-strand cDNA was synthesized using the PrimeScript RT reagent Kit (Takara Bio Inc., Shiga, Japan) with both oligo (dT) primers and random hexamers. Reverse transcription was performed with a Takara PCR Thermal Cycler MP (TP3000). The following are the primer sequences used for amplification of *ITLN*-*1, iNOS, CCL26, periostin, DPP4, CXCL10,* and *GAPDH*: *ITLN*-*1*: forward primer, TGAGGGTCACCGGATGTAAC, reverse primer, GGACTGGCCTCTGGAAAGTA. *iNOS*: forward primer, GACCAGTACGTTTGGCAATG, reverse primer, TTTCAGCATGAAGAGCGATTT. *CCL26*: forward primer, GCTGCTTCCAATACAGCCACA, reverse primer, TCCTTGGATGGGTACAGACTTTC. *periostin*: forward primer, TGTTGCCCTGGTTATATGAGAA, reverse primer, ACATGGTCAATGGGCAAAAC. *DPP4*: forward primer, GCACGGCAACACATTGAA, reverse primer, TGAGGTTCTGAAGGCCTAAATC. *CXCL10*: forward primer, GAAAGCAGTTAGCAAGGAAAGGT, reverse primer, GACATATACTCCATGTAGGGAAGTGA. *GAPDH*: forward primer, GCACCGTCAAGGCTGAGAAC, reverse primer, TGGTGAAGACGCCAGTGGA. *B2M*: forward primer, TTCTGGCCTGGAGGCTATC, reverse primer, TCAGGAAATTTGACTTTCCATTC. *RPLP0*: forward primer, TCTACAACCCTGAAGTGCTTGAT, reverse primer, CAATCTGCAGACAGACACTGG.

The 12.5 µL PCR contained 2 µL of cDNA template, 25 µM in 0.5 µL each forward and reverse primers and 6.25 µL of SYBR Premix Ex Taq (Takara). *GAPDH* was evaluated by using the same PCR protocol as for the interest genes-related pathway elements. DNA was amplified for 40 cycles via denaturation for 5 s at 95 °C and annealing for 30 s at 60 °C, using the Takara Thermal Cycler Dice (TP900). PCR assays were performed and analyzed using the Thermal Cycler Dice Real Time System version 4.2 (Takara Bio Inc). The specificity of the reactions was determined by melting curve analysis. The relative expression of each gene of interest and *GAPDH* were calculated using the ΔΔCt method.

### Correlations between Type-2 related parameters and ITLN-1 expression

FeNO was measured before bronchoscopy at a flow rate of 50 mL/s using the nitric oxide analyzer (NOA) 280i^®^ (Sievers, CO). Correlations between FeNO, serum IgE (measured in the hospital’s clinical lab.) and *ITLN*-*1* mRNA expression in distal and proximal BECs from both ST and SN-Asthma subjects were analyzed. We also measured correlations between *ITLN*-*1* mRNA and *iNOS, CCL26, periostin* and *DPP4* mRNA in distal and proximal BECs from the same subjects.

### Immunohistochemistry

Transbronchial lung biopsies (TBLB) and EBB from SN-Asthma, and ST-Asthma subjects were fixed in formalin. Serial 4 µm sections were immunostained using a rabbit polyclonal antibody against ITLN-1 (1:500) (Abcam, MA) with Dako EnVisionTM FLEX Mini Kit High pH detection system including secondary anti-rabbit antibody for detection. Data were collected using an all-in-one fluorescence microscope, BZ-X700 (KEYENCE, Tokyo, Japan).

### Quantification of ITLN-1 and CXCL10 protein by ELISA

BAL fluid from 18 asthma subjects, 5 ST-Asthma and 13 SN-Asthma (Table 2) and serum from 6 ST-Asthma, and 10 SN-Asthma subjects (Table 3) was collected. There was a little overlap in three study groups. Cell culture supernatants were performed on ALI cultured BECs and HFL-1 cells. ITLN-1 (Immuno-Biological Laboratories Co., Gunma, Japan) or CXCL10 (R&D Systems, Minneapolis, MN) were measured by commercial sandwich ELISAs. Assay ranges are 0.31–20 ng/mL for ITLN-1 and 7.8–500 pg/mL for CXCL10, respectively.

### Culture methods for primary BECs and HFL-1 cells

Freshly isolated BECs were seeded into 60 mm tissue-culture dishes coated with rat-tail type I collagen (BD Discovery Labware, Bedford, MA) in bronchial epithelial growth medium (catalog no. CC-3170, Lonza) in a humidified HEPA-filtered cell culture incubator, supplemented with 5% CO_2_. When the BECs reached 80% confluence, cells were passaged and seeded onto collagen-coated polyester 12-well transwell inserts with BEBM/DMEM. When the cell layer reached 100% confluence in the transwells, the culture method was shifted to the air–liquid interface (ALI) condition by removing the apical medium and maintain this condition for 10 days [4, 23]. BECs were stimulated with or without IL-13 (10 ng/mL), purchased from Peprotech (Rocky Hill, NJ) and Mometasone Furoate (MF) at a concentration of 1 µM (Sigma St Louis, MO).

Human fetal lung fibroblasts (HFL-1; lung, diploid, human, passage 3–7) were obtained from the American Type Culture Collection (Manassas, VA). HFL-1 cells were seeded into 24-well tissue culture plates at a density of 4 × 10^4^ cells/well and cultured at 37 °C in a 5% CO_2_-humidified incubator in Ham’s F12K medium (Sigma, St Louis, MO) containing 10% heat inactivated FBS. Cells were pretreated with recombinant human ITLN-1 (rhITLN-1) (ATGen, Gyeonggi-do, South Korea) at concentrations up to 500 ng/mL for 30 min and then further stimulated with a combination with TNF, IL-1β, and IFN-γ at 10 ng/mL (PeproTech, Rocky Hill, NJ). Cell-culture supernatants and extracts were harvested 24 h later.

### Western blot analysis for phospho-STAT1

Protein samples (10 µg) from HFL-1 were resolved on NuPage Novex 4–12% Bis–Tris gel (Thermo Fisher Scientific, MA) electrophoresis, transferred, and immunoprobed with mouse monoclonal antibody for p-STAT1, total STAT1 (t-STAT1) (1:1000 and 1:500 respectively, Cell Signaling Technologies Inc. MA). Alkaline phosphatase conjugated secondary antibody (Thermo Fisher Scientific) was followed by Chemiluminescent detection (ChemiDoc XRD-J Bio-Rad Laboratories, Inc., CA). Densitometry was performed using the Quantity One (Bio-Rad) and p-STAT1 indexed to t-STAT1.

### Statistical analysis

Variables were checked for normality of distribution. As the majority of data were not normally distributed, data were analyzed using nonparametric tests. The Kruskal–Wallis version of the Wilcoxon rank sum test was used to compare overall differences among the groups (the overall *p* value). When the overall p value was <0.05, intergroup comparisons were done using the Wilcoxon test for multiple comparisons. All other normal distributed data were analyzed using paired t tests compared control and stimulated responses. p values <0.05 were considered significant. Linear regression analysis was used to determine the correlation with Type-2 related parameters and *ITLN*-*1* mRNA. The statistical software used was the JMP version 10 (SAS Institute, Cary, NC).

## Results

### Subjects

Thirteen ST-Asthma, 18 SN-Asthma, 13 D-CON and 17 COPD subjects underwent bronchoscopic airway brushing (Table 1). D-CON and COPD were older than ST or SN-Asthma subjects (*p < 0.0001, ***p < 0.05). FEV1/FVC and  %FEV1 in COPD were lower than in D-CON, ST and SN-Asthma (*p < 0.0001). FeNO was significantly higher in SN-Asthma than the other groups (*p < 0.0001). Mean ICS dose are represented Beclometasone dipropionate (BDP) equivalent dose. Two subjects were using OCS (predonisolone 5 mg/day). Not all subject’s cells were available for every experiments due to the limited numbers of epithelial cells obtained at the time of brushing. Table 2 includes 5 ST and 13 SN-Asthma who underwent BAL. FeNO in SN-Asthma tended to higher than ST-Asthma but this was not significant (p = 0.08). Blood sample was collected from 6 ST-Asthma, 10 SN-Asthma and 8 healthy controls. (Table 3). FeNO in SN-Asthma were significantly higher than SN-Asthma (**p < 0.01).

### *ITLN*-*1* mRNA expression in freshly isolated BECs and correlation with Type-2 related parameters in steroid naïve asthma

**Fig. 1:**
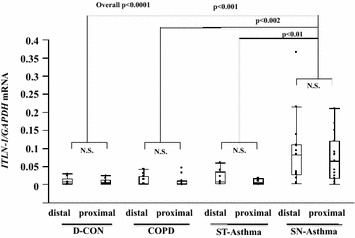
*ITLN*-*1* mRNA expression in freshly isolated BECs from each group by qPCR. *ITLN*-*1* mRNA was significantly enhanced in freshly isolated distal and proximal BECs in SN-Asthma (p < 0.01) compared with other groups. Intergroup comparisons were done using the Wilcoxon test for multiple comparisons

**Fig. 2:**
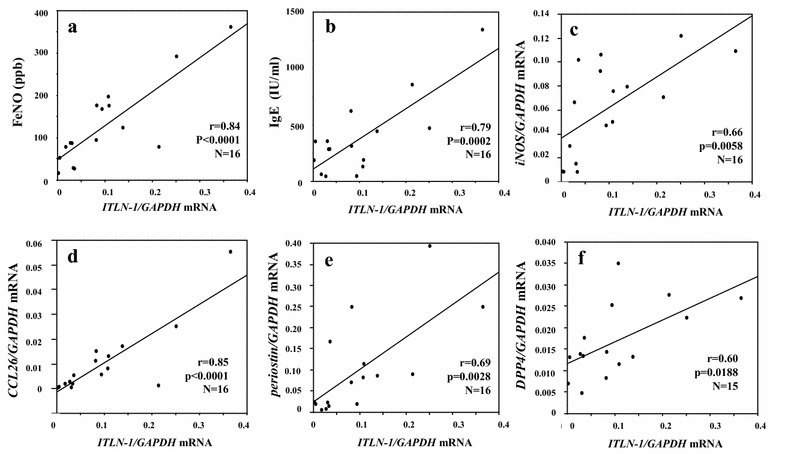
Correlation between *ITLN*-*1* mRNA and Type-2 related parameters in the distal airways in SN-Asthma patients. *ITLN*-*1* mRNA showed significant correlation with FeNO (**a** r = 0.84, p < 0.0001), IgE (**b** r = 0.79, p = 0.0002) *iNOS* (**c** r = 0.66, p = 0.0058), *CCL26* (**d** r = 0.85, p < 0.0001), *periostin* (**e**, r = 0.69, p = 0.0028) and *DPP4* (**f**, r = 0.60, p = 0.0188) mRNA, respectively

The mean counts of freshly isolated BECs from all the subjects were 4.4 ± 0.6 × 10^5^ from distal, and 4.9 ± 1.1 × 10^5^ from proximal (4 brushes each) brushings. They were over 90% pure and 80% viable. *ITLN*-*1* mRNA expression in freshly isolated BECs was significantly higher in the SN-Asthma group than in the other groups in both distal and proximal airway samples (overall p < 0.0001). There were no differences in *ITLN*-*1* mRNA levels between distal and proximal samples among the groups (Fig. 1). Positive correlations were seen between *ITLN*-*1* mRNA expression in the distal BECs and FeNO and IgE in SN-Asthma patients (Fig. 2a: r = 0.84, p < 0.0001, N = 16 and b: r = 0.79 p = 0.0002, N = 16, respectively). *ITLN*-*1* mRNA was also positively correlated with *iNOS*, *CCL26*, *periostin* and *DPP4* mRNA (Fig. 2c–f), respectively. *ITLN*-*1* mRNA and peripheral blood eosinophil numbers were marginally correlated (r = 0.49 p = 0.0556, N = 16, data not shown). In proximal BECs, *ITLN*-*1* mRNA was also correlated with FeNO, *iNOS, CCL26* and *periostin* mRNA. In contrast, in ST-Asthma, *ITLN*-*1* mRNA expression was low and there were no correlations with any Type-2 related parameters (Additional file 1: Table S1).

### ITLN-1 appears to be primarily expressed by goblet cells

**Fig. 3:**
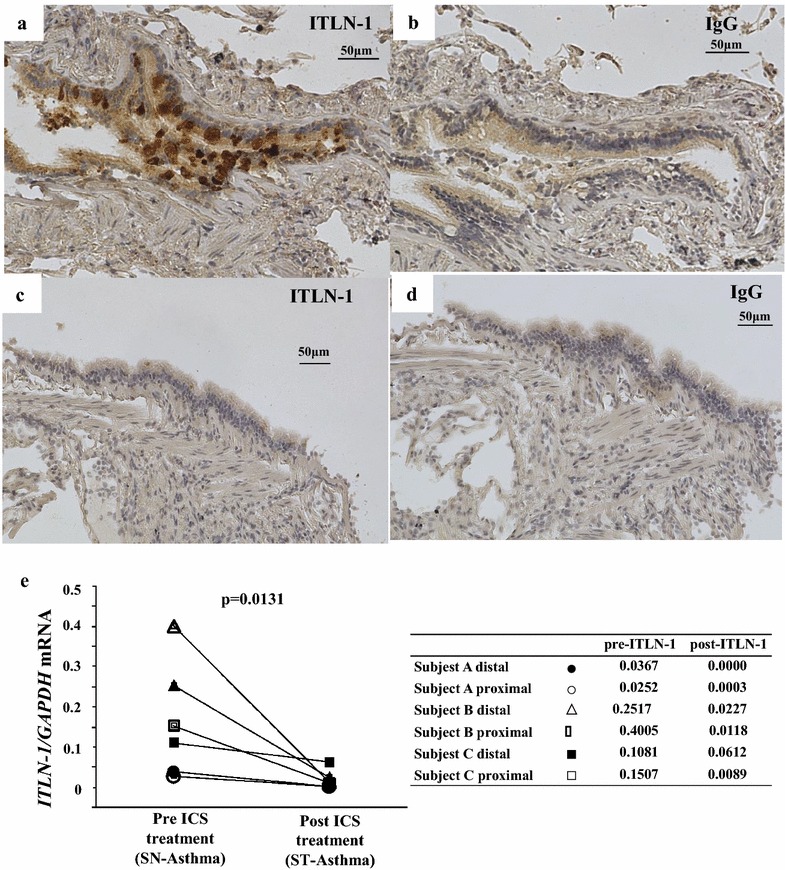
Immunohistochemistry of ITLN-1 expression in airway tissue. Representative distal BECs of SN-Asthma (**a**, **b**), and ST-Asthma (**c**, **d**) samples were stained with anti-ITLN-1 antibody (**a**, **c**) and IgG isotype control (**b**, **d**). The fields are 200 magnificent and antibodies are 1:500 diluted, respectively. ITLN-1 staining is mainly in the goblet cells in SN-Asthma. **e** *ITLN*-*1* mRNA expressions before and after ICS (MF) treatment from 3 patients with 6 samples.* Closed markers* represent distal BECs and* open markers* are proximal BECs. *Circles*, *triangles* and *squares* are represented subjects individually. *Additional small tables* shows *ITLN-1* mRNA values pre and post ICS treatments. Comparisons were done using the Wilcoxon test

Immunostaining of a small number of distal airway biopsy sections indicated that ITLN-1 protein was strongly expressed in goblet cells and weakly in brush border. Figure 3a shows representative staining from 3 SN-Asthma subjects stained with ITLN-1 antibody and isotype control IgG (Fig. 3b). Figure 3c (ITLN-1) and d (IgG) were representative staining from 3 ST-Asthma subjects after ICS (mometasone furoate; MF) treatment. Figure 3c and d was same subject with Fig. 3a, b after ICS treatment. Unfortunately, there were not enough biopsies of sufficient quality to evaluate differences among groups. Figure 3e shows *ITLN*-*1* mRNA expressions in freshly isolated BECs samples in series of before and after ICS (MF) treatment in 6 samples from 3 subjects. Closed markers represent distal BECs and open diamonds are proximal BECs. *ITLN*-*1* mRNA in both distal and proximal BECs significantly decreased after ICS treatments.

### ITLN-1 protein in BAL and serum

**Fig. 4:**
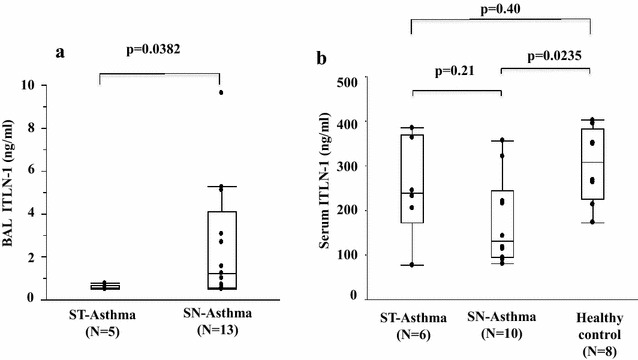
ITLN-1 concentration in BAL and serum. **a** ITLN-1 concentration from BAL samples. ITLN-1 concentration was low (0.5–11.3, mean 2.4 ng/mL) but detectable by ELISA. ITLN-1 is higher in SN-Asthma (n = 13) than in ST-Asthma (n = 5) subjects (p = 0.0382, Wilcoxon test). **b** Serum ITLN-1 concentration is abundant (77.3 to 402 ng/mL, mean 236.5 ng/mL). Serum ITLN-1 is no difference between ST and SN-Asthma subjects (p = 0.21) and ST-Asthma and healthy controls (p = 0.40). However, IT LN-1 is significantly higher than SN-Asthma (p = 0.0235). These comparisons were done using the Wilcoxon test

ITLN-1 was detected in BAL and higher in SN-Asthma than ST-Asthma cases, although the concentrations were very low (Fig. 4a). In contrast, ITLN-1 was easily detected in serum in ST and SN-Asthma cases. Unexpectedly, there was no difference between the ST and SN-Asthma groups (p = 0.21) in Fig. 4b. Serum and BALF albumin concentration were determined by ELISA. The ratio of ITLN-1/albumin was significantly higher in SN-Asthma (Additional file 2: Figure S1).

### *ITLN*-*1* mRNA and protein is induced by IL-13 in primary cultured BECs

**Fig. 5:**
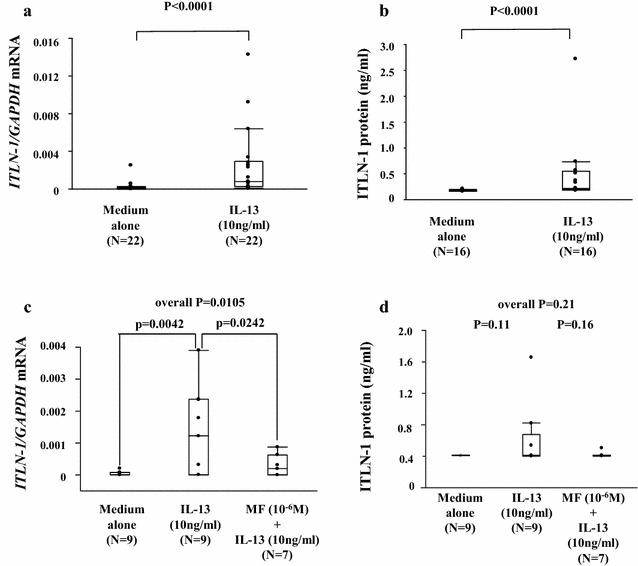
*ITLN*-*1* mRNA expression and protein production induced by IL-13 in primary cultured BECs in vitro. **a** *ITLN*-*1* mRNA expression with or without IL-13 stimulation, in BECs from-asthma patients (p < 0.0001). **b** ITLN-1 protein production enhanced with or without IL-13 stimulation, in BECs from asthma patients (p < 0.0001). **c** Inhibitory effect of MF (1 µM) induced *ITLN*-*1* mRNA expression by IL-13 stimulation asthma subjects. MF significantly decreased *ITLN*-*1* mRNA expression (p = 0.0242). **d** MF inhibited modestly induced ITLN-1 protein production from BECs from asthma subjects. Comparisons were done using the Wilcoxon test

*ITLN*-*1* mRNA expression and protein were measured with or without IL-13 stimulation (10 ng/mL) in primary human BEC derived from both ST and SN-Asthma cultured in ALI. *ITLN*-*1* mRNA expression and protein were significantly enhanced by IL-13 stimulation (Fig. 5a, b). However, amount of *ITLN*-*1* mRNA was very low compared with freshly isolated BECs. Interestingly, ITLN-1 protein was detected only in apical supernatant. There were no differences in *ITLN*-*1* mRNA or protein expression between SN-Asthma and ST-Asthma groups after IL-13 stimulation (Additional file 3: Figure S2a, b). Figure 5c and d show the inhibition effect of MF for induced *ITLN*-*1* mRNA and protein by IL-13. MF inhibited IL-13 induced *ITLN*-*1* mRNA significantly, and modest inhibition effect for ITLN-1 protein. *ITLN-1* mRNA expression was significantly enhanced by IL-13 stimulation normalized by other housekeeping genes (*B2M* and *RPLP0*) (Additional file 4: Figure S3)

### *CXCL10* mRNA and protein expression in HFL-1 cells is inhibited by ITLN-1

**Fig. 6:**
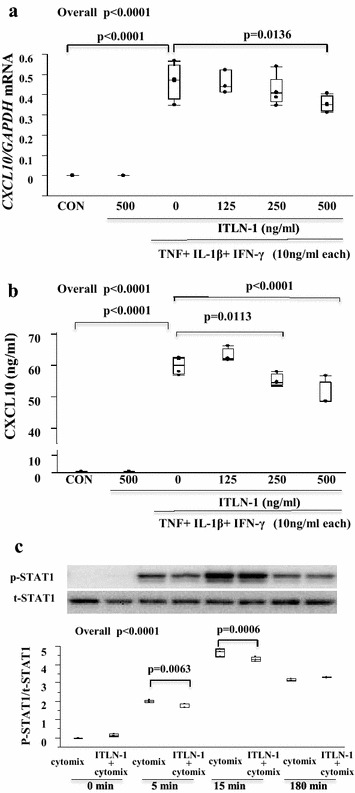
Expression of CXCL10 stimulated by TNF, IL-1β and IFN-γ and inhibition effect by pre-incubated ITLN-1. 30 min pre-incubated by ITLN-1 inhibited expression of *CXCL10* mRNA (**a**) and CXCL10 protein (**b**) in HFL-1 stimulated by cytomix (TNF, IL-1β and IFN-γ, 10 ng/mL each). **a** ITLN-1 at a concentration of 500 ng/mL inhibited cytomix induced *CXCL10* mRNA (p = 0.0136). **b** ITLN-1 at a concentration of 250 and 500 ng/mL inhibited cytomix induced CXCL10 protein (p < 0.0001 and p = 0.0113, respectively). **c** Phospho (p)-STAT1/total (t)-STAT1 level stimulated by cytomix and pre-incubated ITLN-1 in HFL-1. Phospho (p)-STAT1/t-STAT1 level was increased at 5 and 15 min, 30 min pre-treated ITLN-1 was signify inhibited phosphorylation of STAT-1 (p = 0.0006 at 15 min and p = 0.0063 at 5 min)

To determine whether the Type-2 associated ITLN-1 could functionally inhibit Type-1 associated inflammation, CXCL10 expression was induced by the combination of TNF, IL1β and IFN-γ (Cytomix) in HFL-1 cells and the inhibitory effects of ITLN-1 was evaluated (Fig. 6a, b). ITLN-1 (500 ng/mL) alone did not affect CXCL10 expression or production. However, ITLN-1 pretreatment (30 min) reduced Cytomix-induced *CXCL10* mRNA and protein in a concentration-dependent manner. To investigate intracellular signal transduction, we examined STAT1 as a signal transduction pathway of IFN-γ. ITLN-1 decreased cytomix induced p-STAT1 at 5 and 15 min (p = 0.0006, p = 0.0063, respectively), supporting an inhibitory effect on this pathway.

## Discussion

In this study, ITLN-1 was induced by IL-13 and mainly expressed in goblet cells of the distal and proximal airways in SN-Asthma patients. In the SN-Asthma group, *ITLN*-*1* mRNA correlated with FeNO, IgE, *iNOS, CCL26, periostin* and *DPP4* mRNA, all Type-2 related parameters. Finally, our results suggest that ITLN-1 might lead to Type-2-bias by attenuating IFN-γ signaling.

Kupermann et al. reported that ITLN-1 increased in an asthma model and in BECs from asthma subjects [17]. Similar to our data (Fig. 5a, b), Zen et al. showed that ITLN-1 expression increased in NHBE cells stimulated by IL-13 and in the lungs of mice after intranasal IL-13 administration and found ITLN-1 among the induced genes [24]. Gu et al. reported that ITLN-1 is required for expression of IL-13-induced monocyte chemotactic protein (MCP)-1 and -3 in lung epithelial cells and promotes allergic airway inflammation [25]. Thus, ITLN-1 appears to be strongly related to Type-2 inflammation in vitro and in vivo. However, no reports have compared ITLN-1 with other asthma biomarkers or revealed its function in human asthma.

In this study, *ITLN*-*1* mRNA significantly correlated with FeNO and serum IgE, as well as *iNOS, CCL26, periostin* and *DPP4* mRNA in SN-Asthma, as these genes are known to be induced in BECs stimulated with IL-13. Kerr et al. showed that ITLN-1 in sputum is significantly higher in eosinophil-high groups, supporting an association of ITLN-1 with Type-2-high asthma [18]. Immunostaining showed that ITLN-1 protein was expressed in BECs, and suggested it was particularly expressed in goblet cells. The expression levels closely resembled those in intestinal epithelial cells published in previous reports [15, 18].

As Fig. 1 shows, *ITLN*-*1* mRNA expression was significantly higher in freshly isolated BECs from the SN-Asthma group than in the other groups, we hypothesized that ITLN-1 in BAL or more importantly in serum, could be a useful asthma biomarker. Comparing SN-Asthma and ST-Asthma groups only, BAL-ITLN-1 was detected at low levels (range 0.5–9.6 ng/mL), and was significantly higher than in SN-Asthma as compared to ST-Asthma (Fig. 4a). However, BAL fluid collection is invasive, therefore we evaluated serum for ITLN-1. ITLN-1 was abundant in serum (range 77.3–385 ng/mL); but serum ITLN-1 was indistinguishable between ST-Asthma and SN-Asthma patients. This could be because systemic ITLN-1 may originate primarily from the intestinal tract or other organs as opposed to the airways. Moreover, ITLN-1 is expressed in goblet cells and mainly released into the lumens of the airways, such that the amount derived from the airways is not likely to reflect the serum ITLN-1. Thus, it does not appear that serum ITLN-1 will be a valid asthma biomarker.

As described earlier, ITLN-1 is a protective lectin against parasites and microorganisms. Suzuki et al. reported that ITLN-1 is a receptor of lactoferrin which helps to protect against infections [26]. It has been reported that ITLN-1 is expressed in the brush border of intestinal cells and binding of lactoferrin results in activation of signal transduction pathways that control infections. However, data on lactoferrin expression are controversial, with Kerr et al. also reporting increases in lactoferrin asthmatic sputum, while a recent gene array data suggested lower mRNA expression in asthma, particularly Type-2/severe asthma [18, 27]. Thus, further studies are needed to better understand the interactions between ITLN-1 and lactoferrin in asthma.

We also wished to examine the potential functions of ITLN-1 in the airway particularly in relation to Type-1 inflammation. Previously, it was reported that ITLN-1 was one of an adipokine with anti-inflammatory effect [19]. CXCL10 (IP-10) is strongly induced by IFN-γ and is a biomarker of Th1/Type-1 inflammation [28]. We hypothesized that ITLN-1 might skew cellular responses away from Type-1 pathways. Thus, we investigated whether rhITLN-1 could inhibit CXCL10 expression after stimulation with cytomix. ITLN-1 significantly inhibited cytomix induced CXCL10 in HFL-1 cells in a concentration-dependent manner, accompanied by a decrease in phosphorylation of STAT1. These results suggest that ITLN-1 could contribute to a Type-2-high bias in asthmatic airways.

The study limitations include the clinical/observational nature of the study which did not include specific bronchodilator responsiveness testing or methacholine challenge to confirm the asthma diagnosis, particularly in the mild steroid naïve patients. However, despite this lack of objective data, differences in epithelial ITLN-1 expression were apparent on the basis of steroid treatment and in relation to known Type-2 related parameters. We also lacked a true healthy control group. However, it is difficult to perform bronchoscopies in healthy individuals in Japan. Finally, these studies were done as add-on research studies to clinically indicated bronchoscopies in all patients. Therefore, the availability of BAL and tissue samples was limited to a small number of patients.

## Conclusions

ITLN-1 is expressed in untreated asthmatic bronchial epithelial cells, particularly in goblet cells, in association with Type-2 related parameters. However, it appears to be suppressed by corticosteroids in vivo, and epithelial ITLN-1 does not appear to contribute substantially to serum levels, making it unsuitable as a Type-2 asthma biomarker. Its true role in asthma requires further study, perhaps in association with lactoferrin, but it has the potential to further skew inflammation away from Type-1 and towards a Type-2 process.

## Additional files

**Additional file 1: Table S1.** Correlation between *ITLN-1* mRNA and Type-2 related parameters in the SN or ST-Asthma patients.

**Additional file 2: Figure S1.** Serum and BALF albumin concentration and BALF ITLN-1 ratio to BALF albumin.

**Additional file 3: Figure S2.** *ITLN-1* mRNA expression and protein production induced by IL-13 in primary cultured BECs in vitro.

**Additional file 4: Figure S3.** *ITLN-1* mRNA expression normalized by *B2M* and *RPLP0* as housekeeping genes induced by IL-13 in primary cultured BECs in vitro.

## Abbreviations

ITLN-1: intelectin-1
rhITLN-1: recombinant human ITLN-1
BECs: bronchial epithelial cells
FeNO: fractional exhaled nitric oxide
DPP4: dipeptidyl peptidase-4
iNOS: inducible nitric oxide synthase
CCL26: chemokine ligand 26
D-CON: disease control
ICS: inhaled corticosteroid
OCS: oral corticosteroid
SN-Asthma: ICS-naïve bronchial asthma
ST-Asthma: ICS-treated bronchial asthma
TBLB: transbronchial lung biopsy
EBB: endobronchial biopsy
BAL: bronchial alveolar lavage
ALI: air–liquid interface
HFL-1: human fetal fibroblasts
CXCL10: C-X-C motif chemokine 10
STAT1: signal transducer and activator of transcription 1
