# Supplementary figures and images for: Expression of intelectin-1 in bronchial epithelial cells of asthma is correlated with T-helper 2 (Type-2) related parameters and its function
Source: Allergy Asthma Clin Immunol. 2017 Aug 1;13:35. doi: 10.1186/s13223-017-0207-8 (PMC5540302; doi:10.1186/s13223-017-0207-8)

## Slide 1
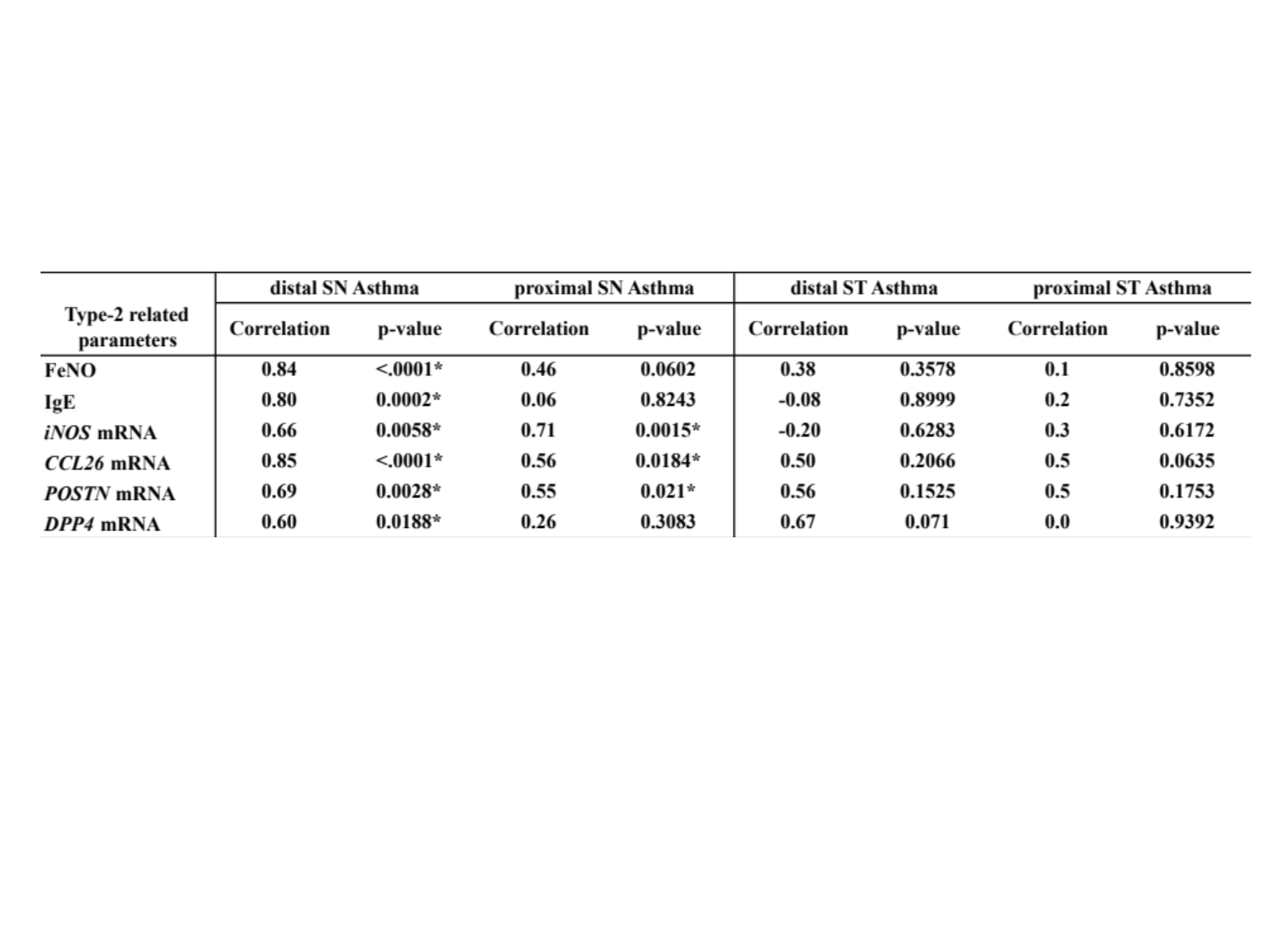

Supplement: Supplementary file 1 — Additional file 1: Table S1. Correlation between ITLN-1 mRNA and Type-2 related parameters in the SN or ST-Asthma patients. [file 13223_2017_207_MOESM1_ESM.pptx]
