# Supplementary material for: Expression of intelectin-1 in bronchial epithelial cells of asthma is correlated with T-helper 2 (Type-2) related parameters and its function
Source: Allergy Asthma Clin Immunol. 2017 Aug 1;13:35. doi: 10.1186/s13223-017-0207-8 (PMC5540302; doi:10.1186/s13223-017-0207-8)
Supplement: Supplementary file 2 — Additional file 2: Figure S1. Serum and BALF albumin concentration and BALF ITLN-1 ratio to BALF albumin. [file 13223_2017_207_MOESM2_ESM.pptx]

## Slide 1
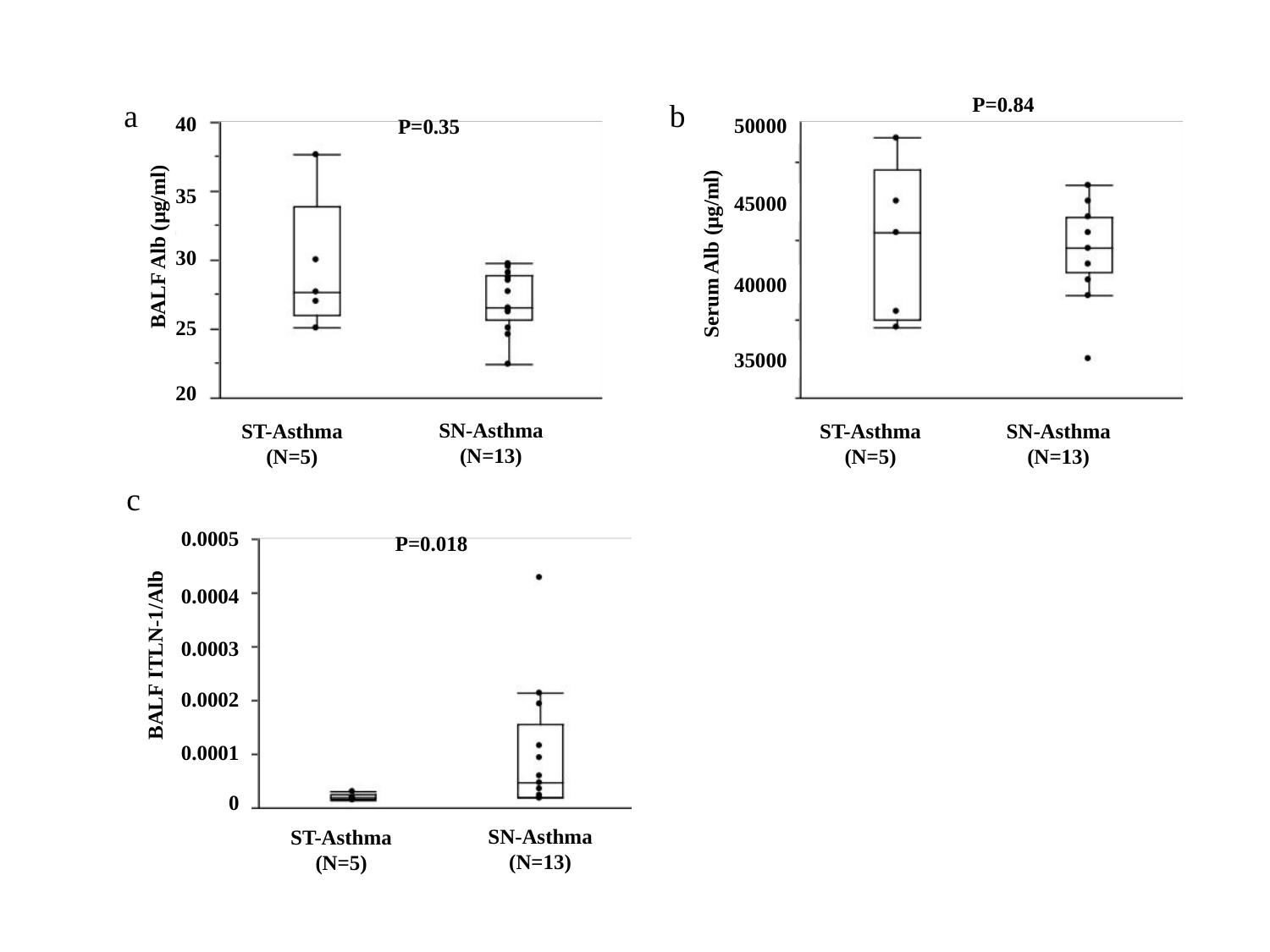

P=0.84
a
b
40
35
30
25
20
50000
45000
40000
35000
P=0.35
Serum Alb (μg/ml)
BALF Alb (μg/ml)
SN-Asthma
(N=13)
ST-Asthma
(N=5)
ST-Asthma
(N=5)
SN-Asthma
(N=13)
c
0.0005
0.0004
0.0003
0.0002
0.0001
0
P=0.018
BALF ITLN-1/Alb
SN-Asthma
(N=13)
ST-Asthma
(N=5)
