# Supplementary material for: Expression of intelectin-1 in bronchial epithelial cells of asthma is correlated with T-helper 2 (Type-2) related parameters and its function
Source: Allergy Asthma Clin Immunol. 2017 Aug 1;13:35. doi: 10.1186/s13223-017-0207-8 (PMC5540302; doi:10.1186/s13223-017-0207-8)
Supplement: Supplementary file 3 — Additional file 3: Figure S2. ITLN-1 mRNA expression and protein production induced by IL-13 in primary cultured BECs in vitro. [file 13223_2017_207_MOESM3_ESM.pptx]

## Slide 1
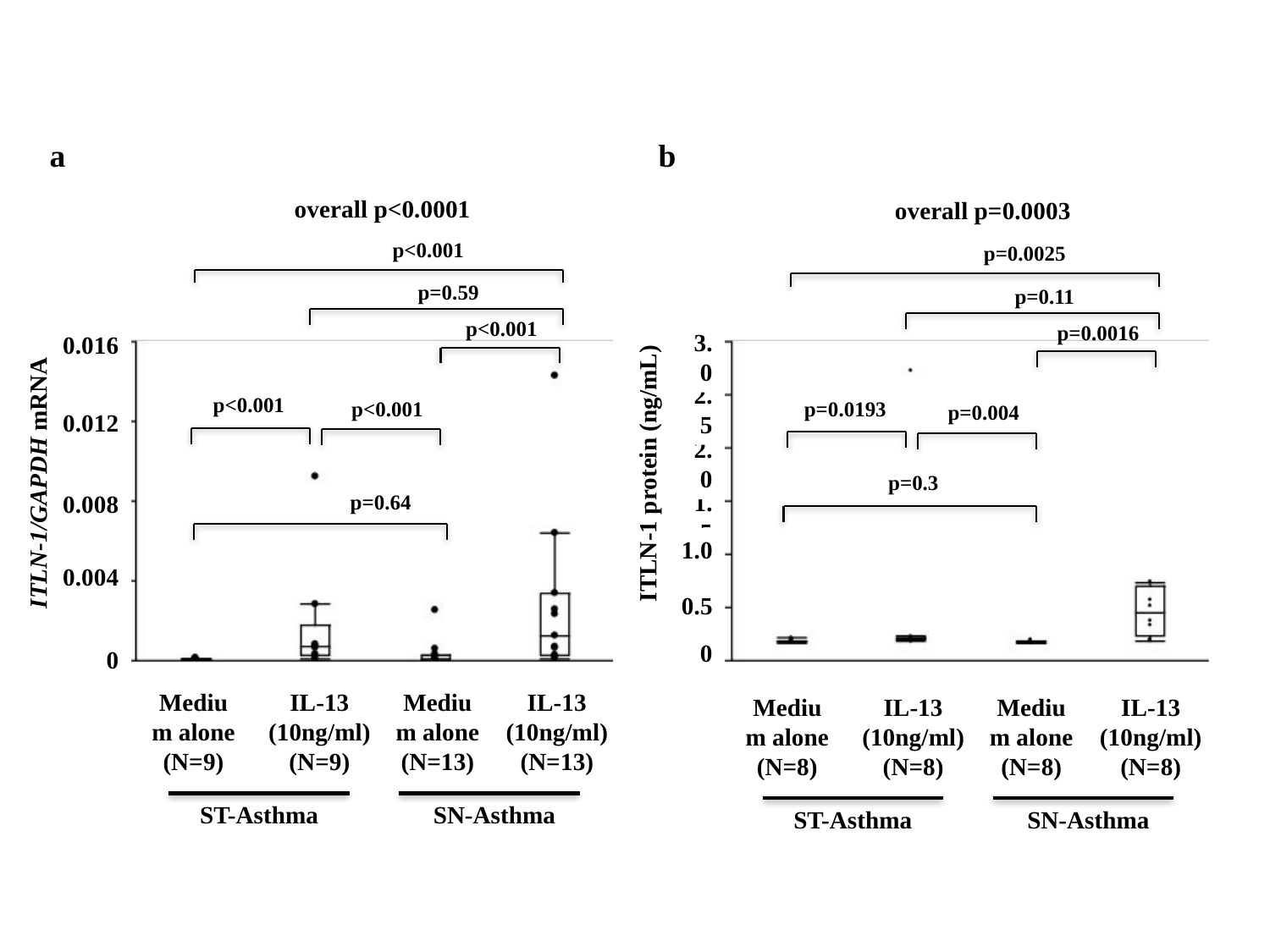

a
b
 overall p<0.0001
 overall p=0.0003
p<0.001
p=0.0025
p=0.59
p=0.11
p<0.001
p=0.0016
3.0
0.016
2.5
p<0.001
p=0.0193
p<0.001
p=0.004
0.012
2.0
ITLN-1 protein (ng/mL)
ITLN-1/GAPDH mRNA
p=0.3
1.5
0.008
p=0.64
1.0
0.004
0.5
0
0
Medium alone
(N=9)
IL-13
(10ng/ml)
(N=9)
Medium alone
(N=13)
IL-13
(10ng/ml)
(N=13)
Medium alone
(N=8)
IL-13
(10ng/ml)
(N=8)
Medium alone
(N=8)
IL-13
(10ng/ml)
(N=8)
ST-Asthma
SN-Asthma
ST-Asthma
SN-Asthma
