# Supplementary material for: Expression of intelectin-1 in bronchial epithelial cells of asthma is correlated with T-helper 2 (Type-2) related parameters and its function
Source: Allergy Asthma Clin Immunol. 2017 Aug 1;13:35. doi: 10.1186/s13223-017-0207-8 (PMC5540302; doi:10.1186/s13223-017-0207-8)
Supplement: Supplementary file 4 — Additional file 4: Figure S3. ITLN-1 mRNA expression normalized by B2M and RPLP0 as housekeeping genes induced by IL-13 in primary cultured BECs in vitro. [file 13223_2017_207_MOESM4_ESM.pptx]

## Slide 1
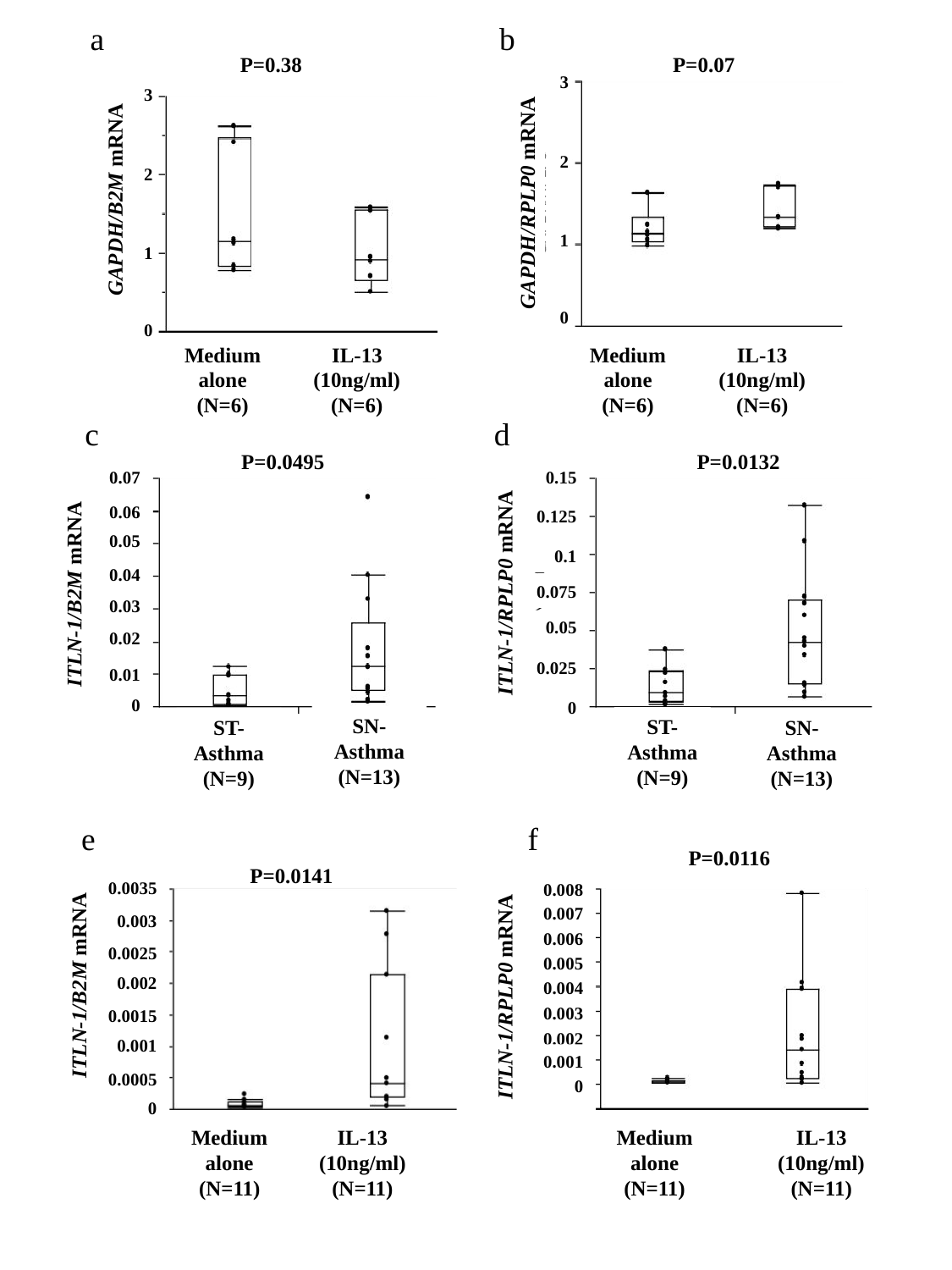

a
b
P=0.38
P=0.07
3
2
1
0
3
2
1
0
GAPDH/B2M mRNA
GAPDH/RPLP0 mRNA
Medium alone
(N=6)
IL-13
(10ng/ml)
(N=6)
Medium alone
(N=6)
IL-13
(10ng/ml)
(N=6)
c
d
P=0.0495
P=0.0132
0.15
0.125
0.1
0.075
0.05
0.025
0
0.07
0.06
0.05
0.04
ITLN-1/RPLP0 mRNA
ITLN-1/B2M mRNA
0.03
0.02
0.01
0
SN-Asthma
(N=13)
ST-Asthma
(N=9)
ST-Asthma
(N=9)
SN-Asthma
(N=13)
e
f
P=0.0116
P=0.0141
0.0035
0.008
0.007
0.006
0.005
0.004
0.003
0.002
0.001
0
0.003
0.0025
0.002
ITLN-1/B2M mRNA
ITLN-1/RPLP0 mRNA
0.0015
0.001
0.0005
0
Medium alone
(N=11)
IL-13
(10ng/ml)
(N=11)
Medium alone
(N=11)
IL-13
(10ng/ml)
(N=11)
